# CAR-Engineered NK Cells Targeting Wild-Type EGFR and EGFRvIII Enhance Killing of Glioblastoma and Patient-Derived Glioblastoma Stem Cells

**DOI:** 10.1038/srep11483

**Published:** 2015-07-09

**Authors:** Jianfeng Han, Jianhong Chu, Wing Keung Chan, Jianying Zhang, Youwei Wang, Justus B. Cohen, Aaron Victor, Walter H. Meisen, Sung-hak Kim, Paola Grandi, Qi-En Wang, Xiaoming He, Ichiro Nakano, E. Antonio Chiocca, Joseph C. Glorioso III, Balveen Kaur, Michael A. Caligiuri, Jianhua Yu

**Affiliations:** 1 Division of Hematology, Department of Internal Medicine, College of Medicine, The Ohio State University, Columbus, Ohio 43210, USA; 2 The Ohio State University Comprehensive Cancer Center, Columbus, Ohio 43210, USA; 3 Center for Biostatistics, The Ohio State University, Columbus, Ohio 43210, USA; 4 Department of Microbiology and Molecular Genetics, University of Pittsburgh School of Medicine, Pittsburgh, PA, 15219 USA; 5 Department of Neurological Surgery, The Ohio State University, Columbus, Ohio 43210, USA; 6 Department of Radiology, College of Medicine, The Ohio State University, Columbus, Ohio 43210, USA; 7 Department of Biomedical Engineering, The Ohio State University, Columbus, Ohio 43210, USA; 8 Department of Neurosurgery, Brigham and Women’s Hospital, Harvard Medical School, Boston, Massachusetts 02115, USA

## Abstract

Glioblastoma (GB) remains the most aggressive primary brain malignancy. Adoptive transfer of chimeric antigen receptor (CAR)-modified immune cells has emerged as a promising anti-cancer approach, yet the potential utility of CAR-engineered natural killer (NK) cells to treat GB has not been explored. Tumors from approximately 50% of GB patients express wild-type EGFR (wtEGFR) and in fewer cases express both wtEGFR and the mutant form EGFRvIII; however, previously reported CAR T cell studies only focus on targeting EGFRvIII. Here we explore whether both wtEGFR and EGFRvIII can be effectively targeted by CAR-redirected NK cells to treat GB. We transduced human NK cell lines NK-92 and NKL, and primary NK cells with a lentiviral construct harboring a second generation CAR targeting both wtEGFR and EGFRvIII and evaluated the anti-GB efficacy of EGFR-CAR-modified NK cells. EGFR-CAR-engineered NK cells displayed enhanced cytolytic capability and IFN-γ production when co-cultured with GB cells or patient-derived GB stem cells in an EGFR-dependent manner. In two orthotopic GB xenograft mouse models, intracranial administration of NK-92-EGFR-CAR cells resulted in efficient suppression of tumor growth and significantly prolonged the tumor-bearing mice survival. These findings support intracranial administration of NK-92-EGFR-CAR cells represents a promising clinical strategy to treat GB.

Glioblastoma (GB) is the most common and the most aggressive primary brain tumor. Even with chemotherapy, radiation, and surgical resection, the median overall survival of GB patients is only 14.6 months^1^. Conventional therapies generally lack specificity and can cause damage to the surrounding brain parenchyma and systemic tissues, a factor that limits their use^2^. Immune-based therapies for GB are a promising alternative to conventional treatments with a potential long-term benefit of generating a sustainable anti-tumor response with potential to target both localized and infiltrating tumor cells^3^.

The epidermal growth factor receptor (EGFR) plays an important role in various tumors including GB. EGFR is the most frequently amplified gene in GB, while its expression in normal brain tissue is either undetectable or extremely low^4^,^5^. Binding of ligand to EGFR leads to receptor homo- and heterodimer formation, autophosphorylation of several key tyrosine residues leading to activation of several intracellular downstream signaling pathways including the Ras/Raf/MEK/ERK pathway, the PLCγ-PKC pathway and the PI3K/AKT pathway, resulting in cell proliferation, motility and survival^6^. Approximately 20–40% of EGFR-amplified tumors harbor the EGFR variant III mutant (EGFRvIII), which contains a deletion of exons 2–7 in the extracellular ligand-binding domain^7^,^8^,^9^,^10^. This mutant form shows constitutive activation in the absence of ligand to activate the tumor-promoting signaling pathways^11^. Collectively, these studies suggest that targeting both wtEGFR and EGFRvIII could be important for effective treatment of GB.

It has been demonstrated that the EGFRvIII-specific CAR-modified T cells exhibited appreciable anti-glioma activity both *in vitro* and *in vivo*^1^,^11^, yet targeting EGFRvIII alone cannot be translated to treat patients with GB expressing wtEGFR, given that the majority of GB patients with EGFR amplification only express wtEGFR^1^,^11^,^12^. As a single agent, EGFRvIII CAR T cells may not effectively treat patients with EGFRvIII-expressing tumor cells, as these patients also have wtEGFR-expressing tumor cells^9^. We thus believe that targeting both forms of EGFR will have a broader application or be more effective than targeting only one. On the other hand, CAR-engineered NK cell lines such as NK-92, which has been used in the clinic^13^,^14^,^15^, could potentially provide an off-the-shelf, renewable product without causing graft-versus-host disease to broadly treat different GB patients. However, use of CAR-modified NK cells in treating GB has not been investigated. In this study, we evaluated the anti-GB capacity of human NK cell lines including NK-92 and NKL and primary NK cells engineered to express an EGFR-specific CAR targeting both wtEGFR and EGFRvIII, and demonstrated the feasibility and efficacy of EGFR-CAR-armed NK cells against GB.

## Results

### Expression of EGFR or EGFRvIII on GB cell lines and patient-derived GB stem cells

**Figure 1:**
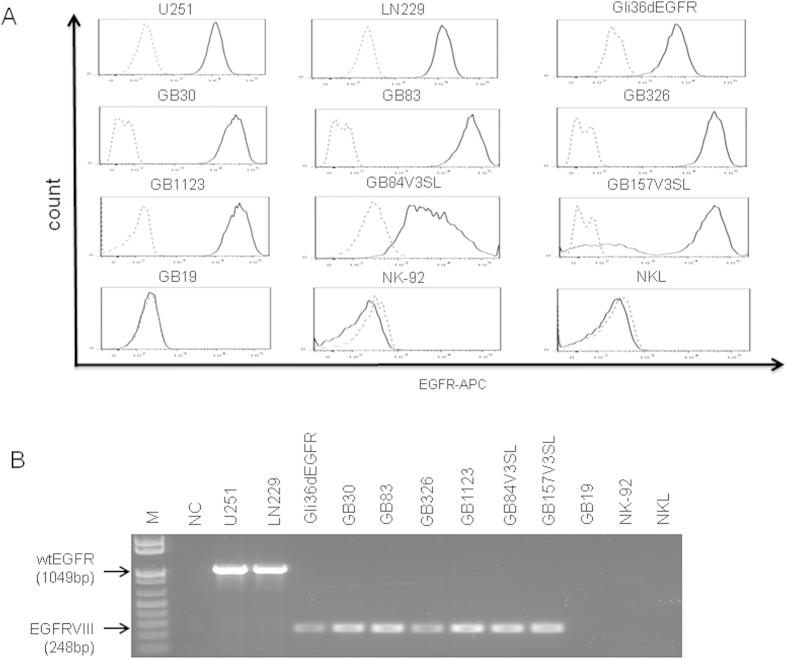
Expression of EGFR on GB and GSCs. (**A**) Surface EGFR expression on glioma cell lines (U251, LN229, and Gli36dEGFR) and GSCs (GB30, GB83, GB326, GB1123, GB84V3SL, GB157V3SL, and GB19) was monitored by flow cytometry. NK cell lines NKL and NK-92 serve as negative controls. (**B**) Determination of EGFR mRNA expression by RT-PCR in glioma cell lines and GSCs specified in **A**. Flow plots and data are representative of three independent experiments.

To assess the surface expression of wtEGFR or EGFRvIII on a panel of GB cell lines and patient-derived GB stem cells (GSCs), intact cells were stained with an EGFR-specific antibody that recognizes both wtEGFR and EGFRvIII, followed by flow cytometric analysis. As shown in Fig. 1A, EGFR was expressed on the surface of GB cell lines (Gli36dEGFR, U251, and LN229), patient-derived mesenchymal (MES) GSCs (GB30, GB83, GB1123 and GB326) and proneural (PN) GSCs (GB84V3SL and GB157V3SL). To further address whether wtEGFR or EGFRvIII transcript was expressed in these cells, we carried out RT-PCR with specific primers and observed that wtEGFR mRNA was expressed in two GB cell lines, U251 and LN229. EGFRvIII mRNA was detectable in the GB cell line Gli36dEGFR, and in GSCs generated from six GB patients^16^: GB30, GB83, GB1123, GB326, GB84V3SL and GB157V3SL (Fig. 1B). In contrast, GSCs from another patient, GB19 (PN), and the two NK cell lines, NK-92 and NKL, had no detectable EGFR expression by flow cytometric analysis or RT-PCR (Fig. 1A,B).

### Generation of NK-92 and NKL NK cells expressing EGFR-CAR

**Figure 2:**
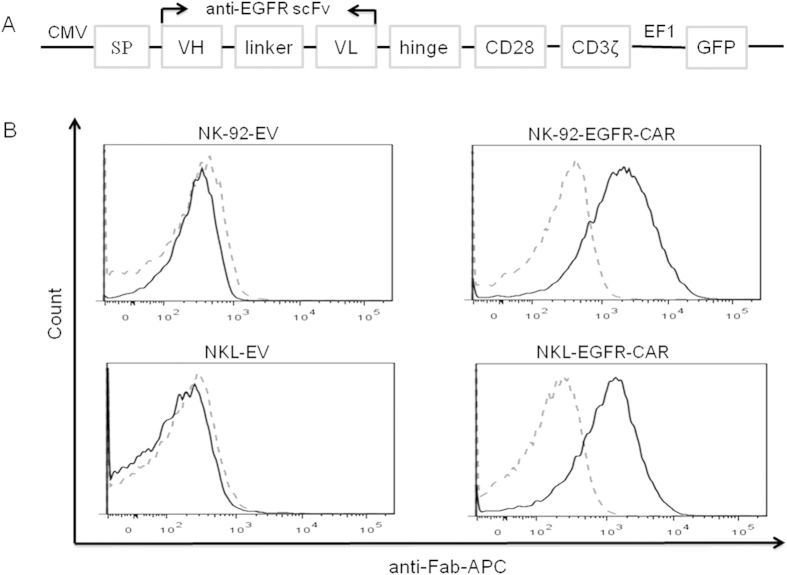
Generation of an EGFR-specific CAR and detection of its expression on CAR-transduced NK cells. (**A**) Schematic representation of the EGFR-CAR lentiviral construct. (**B**) Expression of chimeric EGFR scFv on the surface of NK-92 and NKL cells transduced either with the EGFR-CAR construct (NK-92-EGFR-CAR and NKL-EGFR-CAR, respectively) or the empty vector construct (EV). Cells were FACS-sorted by GFP expression, then analyzed by flow cytometry after the cells were stained with an anti-mouse Fab antibody (solid lines) or IgG isotype control (dashed lines). SP, signal peptide; VH, heavy chain; VL, light chains; scFv, single chain variable fragment. Flow plots and data are representative of three independent experiments.

A second-generation EGFR-specific CAR construct was generated in a pCDH lentiviral vector backbone. This construct sequentially contains a signal peptide (SP), a heavy chain variable region (VH), a linker, a light chain variable region (VL), a hinge, CD28 transmembrane and intracellular domain, and CD3ζ signaling moiety (Fig. 2A). NK-92 and NKL cell lines were transduced with the EGFR-CAR construct to generate NK-92 EGFR-CAR and NKL EGFR-CAR cells, respectively. The transduced cells were sorted for the expression of GFP expressed by the vector. To validate cell surface expression of EGFR-CAR on the transduced NK-92 and NKL cells, we performed flow cytometric analysis using a goat anti-mouse F(ab′)_2_ antibody that recognized the scFv portion of anti-EGFR. The data from Fig. 2B showed an obvious increase in cell surface EGFR-CAR expression in EGFR-CAR-transduced NK-92 and NKL cells over those transduced with empty vector, the latter of which had undetectable EGFR-CAR expression.

### Efficacy of EGFR-CAR-modified NK cell cytotoxicity and IFN-γ production against EGFR^+^ GB cell lines

**Figure 3:**
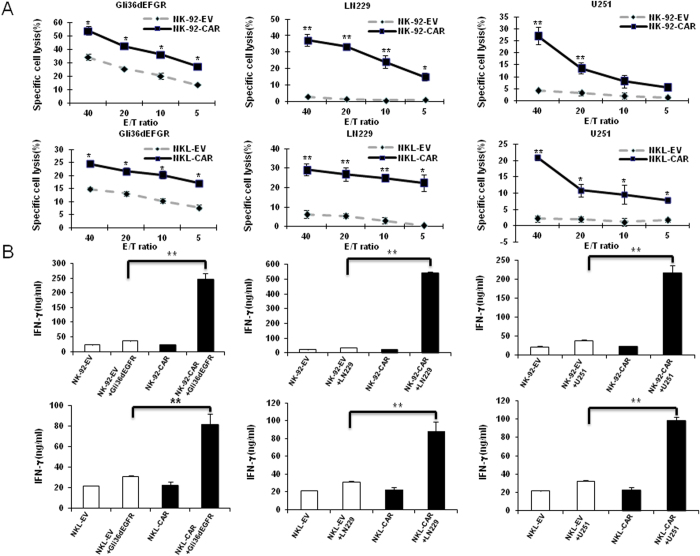
EGFR-CAR-modified NK-92 and NKL cells recognize and kill EGFR^+^ GB cell line cells. **(A**) Cytotoxic activity of empty vector (EV)-transduced or EGFR-CAR-transduced NK-92 and NKL cells against Gli36dEGFR, LN229 or U251 cells using a chromium-51 release assay. (**B**) IFN-γ release of empty vector EV-transduced or EGFR-CAR-transduced NK-92 and NKL cells in the presence or absence of Gli36dEGFR, LN229 or U251 cells using ELISA assay. Representative data of three independent experiments are shown. *p <* *0.05; **p <* *0.01.

We next assessed cytotoxicity of EGFR-CAR- and mock-transduced NK cells against GB cells using a chromium release assay at varying ratios of effector cells to target cells. Figure 3 shows significantly enhanced cytotoxicity of NK-92 EGFR-CAR cells against EGFR^+^ Gli36dEGFR, U251, and LN229 cells compared to control NK-92-EV cells (Fig. 3A**, upper panels**). Similar data were observed in experiments using the NKL cell line transduced with EGFR-CAR (Fig. 3A**, lower panels**). Human primary NK cells transduced with EGFR-CAR also showed significantly more potent cytotoxicity than control cells against EGFR^+^ Gli36dEGFR and U251 GB cell lines (Supplemental Fig. 1A). To determine if the observed enhanced cytolytic activity was accompanied by a similar significant increase in IFN-γ secretion, we co-cultured EGFR-CAR NK-92 cells with EGFR^+^ glioma cells (Gli36dEGFR, U251, and LN229) for 24 h and measured IFN-γ production by ELISA. As shown in Fig. 3B, both EGFR-CAR-modified and mock-transduced NK-92 or NKL cells spontaneously produced low or negligible levels of IFN-γ when incubated alone. Culturing these cells with EGFR^+^ glioma cells (Gli36dEGFR, U251 and LN229) induced IFN-γ in both EGFR-CAR and mock-transduced NK-92 or NKL cell lines, with significantly higher levels of IFN-γ produced by EGFR-CAR-modified NK-92 or NKL cells than by mock-transduced NK-92 or NKL cells, respectively (Fig. 3B). These results are in agreement with the aforementioned cytotoxicity data, and together indicate that modification with EGFR-CAR can significantly enhance NK cell effector functions in response to EGFR^+^ glioma cells.

### Efficacy of EGFR-CAR-modified NK cell cytotoxicity and IFN-γ production against EGFR^+^ GSCs derived from GB patients

**Figure 4:**
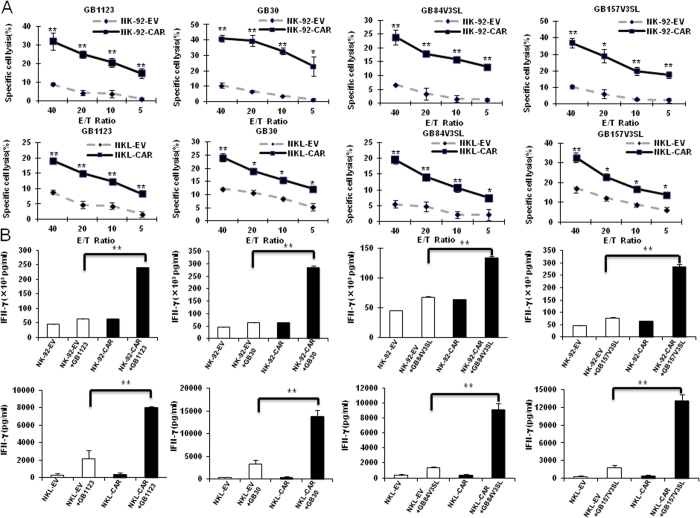
EGFR-CAR-modified NK-92 and NKL cells display enhanced lysis of EGFR^+^ GSCs. (**A**) Cytotoxic activity of NK-92-EV or NK-92-EGFR-CAR cells (upper panel) and NKL-EV or NKL-EGFR-CAR cells (lower panel) against GB1123, GB30, GB157V3SL, and GB84V3SL GSCs using a chromium-51 release assay. (**B)** ELISA analysis of IFN-γ secretion by NK-92-EGFR-CAR or NK-92-EV cells (upper panel) and NKL-EV or NKL-EGFR-CAR cells (lower panel) when co-cultured with GSCs. Representative data of three independent experiments are shown. *p <* *0.05; **p <* *0.01.

We next assessed the capacity of EGFR-CAR-modified NK-92 and NKL cells to lyse patient-derived GSCs with surface expression of endogenous EGFR protein. EGFR-CAR-transduced NK-92 cells demonstrated a significantly enhanced ability to kill EGFR^+^ MES GSCs (GB1123 and GB30) and PN GSCs (GB157V3SL and GB84V3SL) when compared to mock-transduced NK cells (Fig. 4A**, upper panels**). Similar data were observed in experiments repeated using NKL cells transduced with EGFR-CAR (Fig. 4A**, lower panels**). Primary NK cells transduced with EGFR-CAR also showed significantly more potent cytotoxicity than control cells against patient-derived GB30 and GB157V3SL GSCs, which express EGFRvIII (Supplemental Fig. 1B). Likewise, EGFR-CAR-transduced NK-92 and NKL cells produced significantly more IFN-γ when co-cultured with EGFR^+^ GSCs and compared to mock-transduced NK-92 (Fig. 4B **upper panels**) and NKL cells (Fig. 4B **lower panels**). These results indicate that modification of NK cells with an EGFR-CAR can significantly enhance NK cell cytotoxicity and IFN-γ production against EGFR^+^ GSCs compared to unmodified NK cell controls.

### Enhanced cytotoxicity and IFN-γ production of NK-92-EGFR-CAR cells depend on EGFR surface expression on target cells

**Figure 5:**
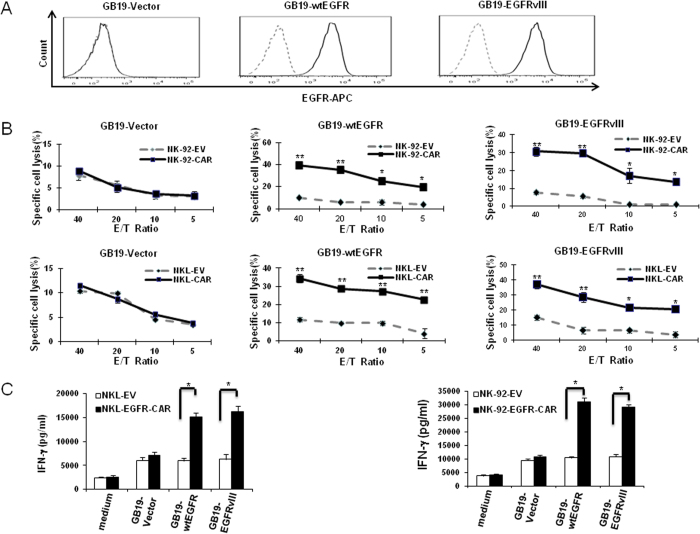
Enhanced target recognition of NK-92-EGFR-CAR cells depends on expression of EGFR on cell surface. **(A**) Flow cytometry using an anti-EGFR antibody (solid line) or IgG isotype control (dotted line) to detect EGFR expression on the surface of GB19 cells transfected with an empty vector (GB19-Vector) or a vector containing wtEGFR (GB19-wtEGFR) or EGFRvIII (GB19-EGFRvIII). (**B**) Cytotoxicity of NK-92-EV or NK-92-EGFR-CAR (upper panel) and NKL-EV or NKL-EGFR-CAR (lower panel) against the GB19-Vector, GB19-wtEGFR, and GB19-EGFRvIII cells shown in **(A)**. The GB19 cells were incubated with NK-92 or NKL cells at various Effector/Target (E/T) ratios for 4* *h. Tumor lysis was determined using a chromium-51 release assay. (**C**) NKL-EV or NKL-EGFR-CAR (left) and NK-92-EGFR-CAR or NK-92-EV cells (right) were co-cultured with equal numbers of GB19-Vector or GB19-wtEGFR or GB19-EGFRvIII cells for 24* *h. Supernatants were then harvested for measurement of IFN-γ secretion using ELISA. Flow plots and data are representative of three independent experiments. *p <* *0.05, **p <* *0.01.

We next explored whether the enhanced cytolytic activity and IFN-γ production in EGFR-CAR-transduced NK-92 or NKL cells triggered by GB cells relies on cell surface EGFR antigen expression. Of the GB cells tested, only GB19 stem cell did not express either EGFR or EGFRvIII on their surface. Thus, we utilized this cell line to investigate whether forced wtEGFR or EGFRvIII overexpression in GB19 cells was sufficient to alter their sensitivity to EGFR-CAR-transduced NK-92 or NKL cells. For this purpose, we overexpressed either wtEGFR or EGFRvIII on GB19 cells by retroviral infection, confirmed by flow cytometric analysis (Fig. 5A). There was a significant increase in the cytotoxic activity of EGFR-CAR-modified NK-92 and -NKL cells towards GB19 cells exogenously overexpressing EGFR or EGFRvIII when compared to target GB19 cells lacking EGFR expression or mock-transduced NK-92 and NKL effector cells (Fig. 5B). Likewise, EGFR-CAR-transduced NK-92 and NKL cells secreted significantly higher levels of IFN-γ when co-cultured with EGFR- or EGFRvIII-overexpressing GB19 cells when compared to target GB19 cells lacking overexpression of EGFR or mock-transduced NK-92 and NKL effector cells (Fig. 5C). These results suggest that the increased recognition and killing of wtEGFR- or EGFRvIII-expressing GB cells by NK-92-EGFR-CAR cells occur in an EGFR-dependent manner. Also, these results were consistent with confirmatory experiments showing that forced EGFR expression in 293T cells resulted in increased cytotoxicity and IFN-γ production of NK-92-EGFR-CAR cells compared to NK-92 cells transduced with the empty vector (Supplemental Fig. 2A–C). Moreover, we pre-treated GB30 and U251 cells with EGFR neutralizing antibody (the same clone as the scFv origin), followed by co-culture of the pre-treated tumor cells with mock-transduced NK-92 cells or NK-92-EGFR-CAR cells. A chromium-51 release assay showed that the EGFR blocking antibody blunted both cytotoxicity and IFN-γ production in NK-92-EGFR-CAR cells when compared to an isotype-matched control antibody (Supplemental Fig. 3), further confirming that the effects that we identified for NK-92-EGFR-CAR cells are EGFR-dependent.

### NK-92-EGFR-CAR cells inhibit GB tumor growth and prolong survival of tumor-bearing mice in two orthotopic xenograft GB models

**Figure 6:**
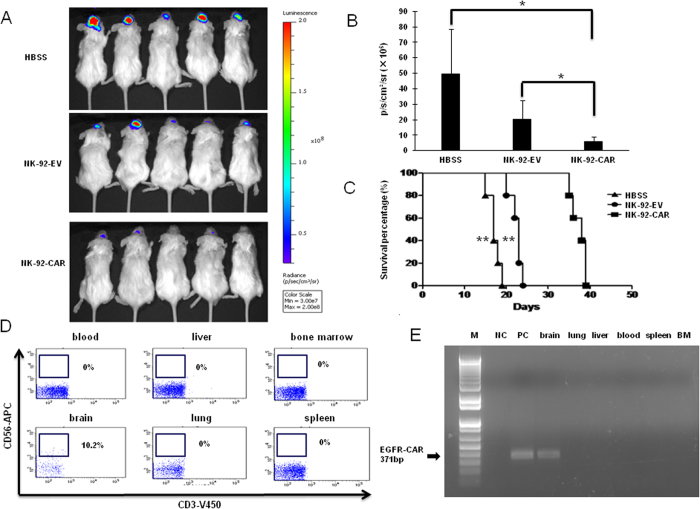
NK-92-EGFR-CAR cells suppress *in vivo* growth of orthotopic human GSCs, prolong the survival of glioma-bearing mice, and localize in the brain without migrating to other organ and tissues. (**A**) Brain bioluminescence imaging of mice bearing GB30 tumors. NSG mice were inoculated with luciferase-expressing GB30 cells via stereotaxic injection (day 0). Seven days after inoculation, mice were intracranially infused once with empty vector-transduced NK-92 cells (NK-92-EV), EGFR-CAR- transduced NK-92 cells (NK-92-EGFR-CAR) or Hank’s buffered salt solution (HBSS; negative control). (**B**) Quantification summary of units of photons per second per mouse from (**A**). * indicates *p *<* *0.05. (**C**) GB30-bearing mice treated with NK-92-EGFR-CAR cells showed significantly increased overall survival compared to the mice treated with NK-92-EV cells or HBSS (** *p *<* *0.01), as determined by Kaplan-Meier survival curves (*n *=* *5 for each group). (**D**) Determination of presence of CD56^+^CD3^-^ human EGFR-CAR NK-92 cells by flow cytometry in liver, lung, blood, spleen, bone marrow (BM), and brain 3 days after intracranial injection of the CAR NK cells into brain of GB30-bearing mice. (**E**) Determination of EGFR-CAR expression by RT-PCR in liver, lung, blood, spleen, bone marrow (BM), and brain 3 days after intracranial injection of the CAR NK cells into brain of GB30-bearing mice. NC* *=* *negative control (no DNA template was added); PC = positive control, EGFR-CAR NK-92 cells. *p <* *0.05, **p <* *0.01.

**Figure 7:**
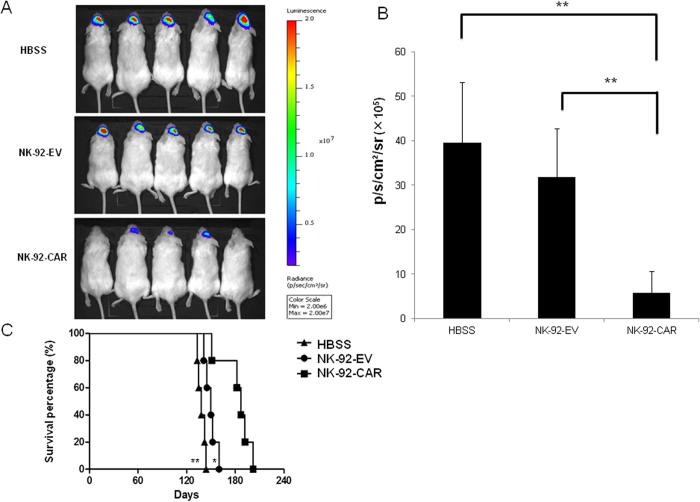
EGFR-CAR-transduced NK-92 cells inhibit wtEGFR-expressing GB tumor growth and prolong survival of tumor-bearing mice in an orthotopic xenograft GB model. (**A**) Brain bioluminescence imaging of mice bearing U251 tumors. NSG mice were intracranially implanted with 10^5^ luciferase-expressing U251 cells via stereotaxic injection (day 0). Days 10, 40, 70 after inoculation, mice were intracranially infused with NK-92-EV cells, NK-92-EGFR-CAR cells or HBSS as negative control. Brain bioluminescence imaging of mice was taken on day 100. (**B**) Quantification summary of units of photons per second per mouse from (**A**). ** indicates *p *<* *0.01. (C) U251-bearing mice treated with NK-92-EGFR-CAR cells showed significantly increased overall survival compared to the mice treated with NK-92-EV cells (* *p *<* *0.05) or HBSS (** *p *<* *0.01), as determined by Kaplan-Meier survival curves (*n *=* *5 for each group).

To further address the potential therapeutic application of NK-92-EGFR-CAR cells, we examined their antitumor activity *in vivo*. We established orthotopic glioma by intracranially implanting EGFRvIII-expressing GB30 GSCs which had been genetically manipulated to express firefly luciferase (GB30-FFL) into the brains of NSG mice. The expression of firefly luciferase enabled us to monitor the tumor growth via *in vivo* bioluminescence imaging. To minimize potential systemic toxicity, we injected the NK-92-EGFR-CAR intratumorally 7 days post tumor cell implantation. As shown in Fig. 6A,B, mice that received either EGFR-CAR- or mock-transduced NK-92 cells had significantly reduced tumor growth as determined by bioluminescence imaging, compared to those injected with Hank’s buffered salt solution (HBSS). Importantly, however, the reduction in tumor growth was significantly greater in mice treated with NK-92-EGFR-CAR cells than those treated with mock-transduced NK-92 cells. In agreement with these data, mice treated with NK-92-EGFR-CAR cells for a single time survived significantly longer than mice treated with mock-transduced NK-92 cells or HBSS (median survival of 38 vs 23 days between NK-92-EGFR-CAR- and NK-92-EV-treated mice, *p *<* *0.01; median survival of 38 vs 17 days between NK-92-EGFR-CAR- and HBSS-treated mice, *p *<* *0.01) (Fig. 6C). To further address the therapeutic efficacy of NK-92-EGFR-CAR cells against wtEGFR-expressing GB tumor, we established an orthotopic GB model by intracranially implanting wtEGFR-expressing U251-FFL cells into NSG mice. We injected NK-92-EGFR-CAR cells, NK-92-EV cells or HBSS as a vehicle control intratumorally 10 days, 40 days and 70 days after tumor cell implantation. As shown in Fig. 7A,B, mice with a single intratumoral injection of EGFR-CAR cells had significantly decreased tumor burden, compared to those infused with HBSS or NK-92-EV cells. Moreover, mice treated with NK-92-EGFR-CAR cells survived significantly longer than those receiving NK-92-EV cells or HBSS (median survival of 187 vs 150 days between NK-92-EGFR-CAR- and NK-92-EV cell-treated mice, *p *<* *0.05; median survival of 187* *vs 138 days between NK-92-EGFR-CAR- and HBSS-treated mice, *p *<* *0.01) (Fig. 7C). Taken together, NK-92-EGFR-CAR cells could efficiently target and kill either wtEGFR- or EGFRvIII-expressing GB *in vivo*.

### Assessment of NK-92-EGFR-CAR cell migration after intracranial injection

To evaluate the safety of intracranial injection of NK-92-EGFR-CAR cells, we first analyzed the systemic cell distribution after intracranial injection. We undertook a flow cytometric analysis and a more sensitive PCR approach to assess the distribution of NK-92-EGFR-CAR cells in a variety of organs and tissues harvested three days after their intracranial injection into GB30-bearing mice. As shown in Fig. 6D, CD56+ cells could be identified only in the brain and constituted only 10.2% of total immune infiltrating cells in the brain. Similarly, PCR analysis was unable to detect EGFR-CAR expression in any organ site tested other than brain (Fig. 6E). We next determined whether intratumorally infused NK-92-EGFR-CAR cells migrated outside the tumor into normal brain tissue. For this purpose, we performed Hematoxylin and Eosin (H&E) staining of brain sections of GB30-bearing mice being intratumorally infused with NK-92-EGFR-CAR cells. The results showed that NK-92-EGFR-CAR cells distributed only inside the tumor (Supplemental Fig. S4). To determine whether NK-92-EGFR-CAR cells cause damage to brain tissue, NK-92-CAR cells were intracranially injected into NSG mice without tumor. Cleaved caspase-3 IHC staining of brain tissue from the treated mice showed that majority of apoptotic cells seem to be NK-92-EGFR-CAR cells (Supplemental Fig. S5B) and some host cells in the tissue damaged by a needle injection (Supplemental Fig. S5C). Nearly no damages by NK-92-EGFR-CAR cells were observed in other areas (Supplemental Fig. S5D and S5E) when compared with brain tissue harvested from un-treated control mice (Supplemental Fig. S5F). Together, these data suggest that intracranial administration of NK-92-EGFR-CAR cells for the treatment of GB is a safe approach.

## Discussion

In this study, we developed a novel and promising strategy against GB, utilizing EGFR-CAR-modified human NK-92 cells to intracranially target human GB. Tumors from GB patients express either wtEGFR or both wtEGFR and EGFRvIII^9^, suggesting that targeting both forms of this surface protein will have a broader application or be more effective than targeting only one. We also demonstrated the *in vivo* efficacy and safety of intracranial injection of EGFR-CAR-modified NK-92 cells in our orthotopic preclinical model.

CAR T cells have been effectively used for treatment of refractory chronic lymphocytic leukemia and acute lymphoblastic leukemia, and represent a powerful new therapeutic modality for these highly drug-resistant tumors^17^,^18^,^19^,^20^. Also, several studies have demonstrated the use of CAR T cells to treat GB^1^,^11^,^21^. But these studies only focused on targeting EGFRvIII. Moreover, CAR T cells can cause cytokine-related adverse events and tumor lysis syndrome^19^,^22^, which may result in substantial toxicity or death of patients. In addition, production of autologous CAR T cells is an expensive and time-consuming approach. Thus, utility of CAR NK cells or CAR NK cell line cells to target both wtEGFR and EGFRvIII for GB treatment is a good alternative approach.

CAR-engineered NK cell lines (e.g. NK-92 used in this study) could potentially provide an off-the-shelf, renewable product to broadly treat different GB patients. Our present study is the first to investigate the utilization of CAR-modified NK-92 cells against GB. CAR-modified NK-92 cells have been shown to effectively target other cancers. For example, Esser *et al*. engineered NK-92 cells to stably express CAR against disialoganglioside GD2, which is expressed at high levels on neuroblastoma (NB) cells^23^. Expression of GD2-CAR on NK-92 cells enhanced specific elimination of GD2-expressing NB cells that were resistant to killing by parental NK-92 cells, demonstrating the potential clinical utility of the GD2-CAR NK cells against NB. We have recently developed CS1-CAR-expressing NK-92 cells to target multiple myeloma (MM) cells and demonstrated that these engineered NK-92 cells were able to display enhanced cytolysis and IFN-γ production when co-cultured with MM cells *in vitro*^24^. Importantly, in an aggressive orthotopic MM xenograft mouse model, adoptive transfer of CS1-CAR-modified NK-92 cells efficiently impeded the dissemination of human IM9 MM cells *in vivo* and also significantly prolonged the overall survival of IM9-bearing mice 24.

To our knowledge, this is the first study investigating the potential of CAR immune cells to target both wtEGFR and EGFRvIII for GB treatment. We generated EGFR-CAR-engineered NK-92 and NKL cells as well as primary NK cells which efficiently lyse GB cells expressing either wtEGFR or EGFRvIII *in vitro*. Both molecules play contributory roles in gliomagenesis. EGFRvIII-expressing cells typically co-exist with wtEGFR-expressing cells in same patients. Biernat reported that 40% (8 out of 20) of EGFR^+^ GB biopsies also showed positive for EGFRvIII, but only 15% (3 out of 20) with a predominant EGFRvIII amplification, suggesting that the limited effectiveness could only be achieved for therapeutic approaches based on selective targeting of EGFRvIII^8^. In another study, among 58* *GB tumors, 83% (48/58) stained for wtEGFR by IHC. Furthermore, 19% (11/58) of these samples were double positive for wtEGFR and EGFRvIII.^9^ These two studies demonstrate that a substantial portion of GB patients have both wtEGFR-expressing and EGFRvIII-expressing tumor cells. Thus, compared with therapeutic approaches to treat GB selectively targeting either wtEGFR or EGFRvIII, the EGFR-CAR NK therapy described here targets both wtEGFR and EGFRvIII. As noted above, EGFRvIII-specific CAR T cells show good anti-tumor activity against GB expressing EGFRvIII both *in vitro* and *in vivo*^1^,^11^,^12^. We believe that an approach targeting both wtEGFR and EGFRvIII should be effective to treat not only GB patients with wtEGFR-expressing tumor cells alone, but also GB patients with both wtEGFR-expressing tumor cells and EGFRvIII-expressing tumor cells. For this purpose, we engineered NK-92 cells to express an EGFR-CAR targeting both wtEGFR and EGFRvIII, and demonstrated that GB cells expressing wtEGFR or EGFRvIII could be efficiently eliminated *in vitro* and *in vivo*.

A potential challenge of targeting wtEGFR as well as EGFRvIII on glioma cells is the presence of wtEGFR on normal tissues. Systemic infusion of EGFRvIII-specific CAR T cells is believed to be relatively safe, as EGFRvIII is not expressed on normal epithelial cells that can express high levels of wtEGFR. However, systemic administration of NK-92-EGFR-CAR or primary NK cells could result in serious systemic toxicity due to their impact on normal wtEGFR-expressing cells. To minimize this risk, we injected NK-92-EGFR-CAR cells intracranially to restrict their mobility, and demonstrated that these CAR cells remained undetectable in tissues other than brain. In further support of this strategy, a recent study shows that intracranial delivery of toxin-conjugated scFv from an EGFR antibody targeting both wtEGFR and EGFRvIII results in strong anti-neoplastic effects against intracranial glioblastoma xenografts expressing wtEGFR or co-expressing wtEGFR and EGFRvIII. This local administration did not cause obvious systemic toxicity^25^. In addition, we performed H&E staining and found that NK-92-EGFR-CAR cells injected into implanted tumors in the brain resided in the tumor and exhibited negligible damages to the surrounding brain tissue. However, as NK-92 is a lymphoma cell line, for future clinical applications, NK-92 cells armed with a CAR should be irradiated prior to infusion into patients. Several preclinical studies demonstrated that NK-92-CAR cells receiving 10 Gy of radiation showed similar effects *in vitro* and *in vivo* for a period of time when compared with non-irradiated cells^26^,^27^. Alternatively, EGFR-CAR primary NK cells should be considered, as we showed that they also potently lysed GB cells and as genetically-engineered artificial antigen-presenting cells (aAPCs) expressing membrane-bound IL-15 (mbIL15) or membrane-bound IL-21 (mbIL21) have recently been used to successfully expand primary NK cells^28^,^29^.

Importantly, in our study we observed high expression of endogenous EGFRvIII on the majority of patient-derived GSCs tested, making them more susceptible to lysis by NK-92-EGFR-CAR cells than by NK-92 cells transduced with control vector. GSCs are a subpopulation of tumor cells that reflect biological and pathological characteristics of primary GB, and retain the capability to undergo self-renewal, multi-lineage differentiation, and regeneration of the entire tumor population^28^. GSCs are considered to be responsible for tumor initiation, propagation, recurrence, and chemo- and radio-resistance. Targeting GSCs is believed to be a key aspect in the prevention of GB relapse. In fact, our *in vivo* data show that one time administration of NK-92-EGFR-CAR cells significantly prolongs the survival of mice implanted with patient-derived GSCs expressing EGFRvIII. Thus, we believe our study demonstrating the use of NK-92-EGFR-CAR cells to target GB cells and GSCs both *in vitro* and in two xenograft mouse models is highly relevant to the potential treatment of relapsed human GB in the clinic.

GB is composed of distinct molecular subtypes. MES and PN GBs have been identified as the most important subtypes of GB^16^,^29^,^30^,^31^. In our experiments, all MES and two of three PN GSCs that we tested were positive for EGFR expression by flow cytometry. PCR analysis further demonstrated that all of these GSCs express EGFRvIII, while GB non-stem cell lines usually express only wtEGFR (except for ectopic over-expression of EGFRvIII in Gli36dEGFR). Consistent with these findings, our *in vitro* data demonstrate that EGFR-CAR NK cells display enhanced IFN-γ secretion and cytotoxicity when cultured with either PN or MES EGFR^+^ GSCs *in vitro*. MES GSCs are more aggressive *in vitro* and are capable of quickly giving rise to intracranial xenografts *in vivo*^16^. MES GSCs also exhibit remarkable resistance to radiation compared with PN GSCs^16^. Our *in vivo* data demonstrated that the NK-92-EGFR-CAR cells showed efficacy in the aggressive GB30 MES GSC xenograft model.

In conclusion, we have successfully generated CAR NK cells that target both wtEGFR and EGFRvIII in GB. NK cells armed with this EGFR-CAR efficiently and specifically recognize and kill GB cells and patient-derived GSCs *in vitro* and *in vivo*. Our study supports the clinical application of EGFR-CAR-modified NK-92 cells for the treatment of relapsed GB, which may be locally administered alone or in combination with other approaches.

## Methods

### Cell culture

Human GB cell lines [Gli36DeltaEGFR (Gli36dEGFR), U251, and LN229] and human patient-derived GSCs (GB1123, GB30, GB83, and GB326)^16^ were used in this study. GB84V3SL, GB157V3SL, and GB19 were also established from GB patients using the previously described protocol^16^. All GB cell lines, 293T cells and Phoenix cells were cultured in DMEM (Invitrogen, Grand Island, NY) supplemented with 10% FBS, penicillin (100 U/ml), and streptomycin (100* *μg/ml). GSCs were cultured in DMEM-F-12 medium supplemented with bFGF (20* *ng/ml), EGF (20* *ng/ml), Glutamax (1:100), B27 (1:100), heparin (5 μg/ml), penicillin (100 U/ml), and streptomycin (100* *μg/ml). All stocks of the above antibiotics and cytokine stocks were purchased from Invitrogen. Human NK cell lines NK-92 and NKL were maintained in RPMI-1640 (Invitrogen) supplemented with 20% FBS, penicillin (100* *U/ml), streptomycin (100* *μg/ml) and 150* *IU/mL recombinant human (rh) IL-2 (Hoffman-La Roche Inc., Nutley, NJ).

### Mice

Six to eight-week-old NOD.Cg-*Prkdc*^*scid*^ *Il2rg*^*tm1Wjl*^/SzJ mice (NSG) mice were obtained from Jackson Laboratories (Bar Harbor, ME). All animal work was approved by The Ohio State University Animal Care and Use Committee and the methods were carried out in accordance with the approved guidelines. Mice were monitored frequently for GB disease progression, and sacrificed when they became moribund with neurologic impairments and obvious weight loss.

### Generation of anti-EGFR CAR lentiviral construct

The anti-EGFR single chain variable fragment (scFv) was derived from a hybridoma cell line that produces mouse monoclonal antibody 528 recognizing both wtEGFR and EGFRvIII^32^,^33^. The coding domain sequences for variable regions of heavy (VH) and light (VL) chains were amplified separately and assembled using a linker by overlapping PCR reaction. The VH-linker-VL fragment was incorporated in frame with CD28-CD3ζ portion^34^ that was incised from a retroviral vector. The entire anti-EGFR-scFv-CD28-CD3ζ fragment was then ligated into a lentiviral vector designated pCDH-CMV-MCS-EF1-copGFP (pCDH, System Biosciences, Mountain View, CA) to generate a pCDH-EGFR-scFv-CD28-CD3ζ (pCDH-EGFR-CAR) construct.

### Lentivirus production and transduction

To produce lentiviruses for infection of NK cell lines (NK-92 and NKL), 293T cells were co-transfected with the aforementioned pCDH-EGFR-scFv-CD28-CD3ζ plasmid or a mock pCDH vector together with the packaging constructs pCMV-VSVG and pCMV-DR9 using calcium phosphate transfection reagent (Promega, Madison, WI). The transfection and infection procedures were modified from a previously published protocol^24^.

### Generation of GB19 stem cells stably expressing wtEGFR or EGFRvIII

Phoenix cells were co-transfected with the pBABE-wtEGFR (a wtEGFR expression construct), or pBABE-EGFRvIII (an EGFRvIII expression construct) or a pBABE empty vector together with Sara3 packaging plasmid using calcium phosphate transfection reagent (Promega, Madison, WI). Two days after transfection, the infectious supernatants were harvested to infect GB19 stem cells in the presence of polybrene (8 μg/mL). Retroviruses were produced in serum-free DMEM-F12 medium for infection of GB19 stem cells, and GFP positive GB19-wtEGFR or GB19-EGFRvIII cells were then sorted using a FACS Aria II cell sorter (BD Biosciences, San Jose, CA).

### Flow cytometry

To evaluate NK cell surface expression of EGFR-CAR, transduced NK cells were washed with PBS containing 4% BSA, and incubated with biotin-labeled goat anti-mouse F(ab′)_2_ polyclonal antibody or normal polyclonal goat IgG antibody (Jackson ImmunoResearch, West Grove, PA) as an isotype control as described previously^24^. Then cells were washed again and stained with allophycocyanin (APC)-conjugated streptavidin (Jackson ImmunoResearch, West Grove, PA). To determine wtEGFR and/or EGFRvIII expression on the surface of GB cells, the cells were incubated with mouse monoclonal anti-human EGFR (clone H11, DAKO, Carpinteria, CA) which recognizes both wild-type EGFR and its mutant form (EGFRvIII), followed by staining with APC conjugated goat anti-mouse IgG second antibody.

### Reverse transcription PCR

To detect EGFR mRNA expression in glioma cells, RNA was extracted from the cell lines with RNeasy Mini Kit (Qiagen, Hilden, Germany) and quantified with NanoDrop (Thermo Fisher, Wilmington, DE). Reverse transcripts were produced using M-MLV reverse transcriptase (Invitrogen, Grand Island, NY), and PCR was conducted with GoTaq® Flexi DNA Polymerase (Promega, Madison, WI). The forward primer is TGACTCCGTCCAGTATTGATCG, and the reverse primer is GCCCTTCGCACTTCTTACACTT. The PCR reaction parameters are 95* *°C 5 min, 35* *cycles at 95* *°C 40* *s, 55 °C 40* *s, 72 °C 1 min, and final extension at 72* *°C for 10* *min.

### Cytotoxicity assay

A standard 4-h ^51^Cr release assay was performed as described previously^35^. Briefly, target cells were labeled with ^51^Cr and co-cultured with modified NK cells at various effector/target ratios (E/T) in the wells of 96-well V-bottom plates at 37* *°C for 4* *h. Supernatants were harvested and transferred into scintillation vials containing a liquid scintillation cocktail (Fisher Scientific, Waltham, MA), and the release of ^51^Cr was measured on Beckman Liquid Scintillation Counter LS-6500. Target cells incubated in complete medium or 1% SDS were used to determine spontaneous or maximal ^51^Cr release, respectively. Percentage of specific cell lysis was calculated using the standard formula: 100 x (cpm experimental release – cpm spontaneous release) / (cpm maximal release – cpm spontaneous release).

### IFN-γ release assay

1* *×* *10^6^ target cells were incubated with equal numbers of NK effector cells in 96-well V bottom plates for 24* *h. Cell-free supernatants were assayed for IFN-γ secretion by enzyme-linked immunosorbent assay (ELISA) using a kit from R&D Systems (Minneapolis, MN) in accordance with the manufacturer’s protocol. Data depicted in figures represent mean values of triplicate wells from one of three representative experiments with similar results.

### Treatment of orthotopic human GB30 xenografts in NSG mice

GB30 GSCs were retrovirally transduced with Pinco-pGL3-luc/GFP virus expressing firefly luciferase (FFL) as previously described^36^. GFP positive cells were sorted using a FACS Aria II cell sorter (BD Biosciences, San Jose, CA), and were designated “GB30-FFL” cells. NSG mice were anesthetized and fixed in a stereotactic apparatus, a burr hole was drilled 2* *mm lateral and 1* *mm anterior to the bregma to a depth of 3.25* *mm, and 5* *×* *10^4^ GB30-FFL cells in 2* *μl HBSS were implanted. On day 7, the mice were intracranially injected with 2* *×* *10^6^ effector cells (non-irradiated), i.e. EGFR-CAR-transduced NK-92 cells (NK-92-EGFR-CAR) or mock-transduced NK-92 cells (NK-92-EV) in 5* *μl HBSS. Mice treated with 5* *μl HBSS only were used as control. Mice were monitored daily and euthanized when showing signs of morbidity. Two weeks after GB30-FFL cell inoculation, the mice were intraperitoneally (i.p.) infused with D-luciferin (150* *mg/kg body weight; Gold Biotechnology, St. Louis, MO), anesthetized with isoflurane, and imaged using *In Vivo* Imaging System (IVIS-100, PerkinElmer, Waltham, MA) with living image software (PerkinElmer).

### Treatment of orthotopic human U251 xenografts in NSG mice

U251 GB cells were retrovirally transduced with Pinco-pGL3-luc/GFP virus expressing firefly luciferase (FFL) as previously described 36. GFP positive cells were sorted using a FACS Aria II cell sorter (BD Biosciences, San Jose, CA), and were designated “U251-FFL” cells. NSG mice were anesthetized and fixed in a stereotactic apparatus, and a burr hole was drilled 2* *mm lateral and 1* *mm anterior to the bregma to a depth of 3.25* *mm, through which 10^5^ U251-FFL cells in 2* *μl HBSS were inoculated on day 0. On days 10, 40, 70 the mice were intracranially injected with 2* *×* *10^6^ effector cells, i.e. EGFR-CAR-transduced NK-92 cells (NK-92-EGFR-CAR) or mock-transduced NK-92 cells (NK-92-EV) in 5* *μl HBSS. Mice treated with 5* *μl HBSS only were used as control. Mice were monitored daily and euthanized when showing signs of morbidity. On day 100 after U251-FFL cell inoculation, the mice were intraperitoneally (i.p.) infused with D-luciferin (150* *mg/kg body weight; Gold Biotechnology, St. Louis, MO), anesthetized with isoflurane, and imaged using *In Vivo* Imaging System (IVIS-100, PerkinElmer, Waltham Massachusetts, USA) with living image software (PerkinElmer).

### Organ and tissue distribution assays of NK-92-EGFR-CAR cells

2* *×* *10^6^ NK-92 EGFR-CAR cells were intracranially injected into three GB30-bearing mice 7 days after tumor implantation. Three days later, all mice were sacrificed and liver, lung, spleen, bone marrow, blood, and brain were harvested. Half of organs or tissues were used for genomic DNA isolation with a DNA isolation kit (Invitrogen, Carlsbad, CA), and PCR was performed with primers to amplify the EGFR-CAR scFv region. The PCR forward primer was AGGTCACTCTGACTGTAGACA, and the reverse primer was GTTCATGTAGTCACTGTGCAG. The PCR reaction parameters were the same as those described above.

The other half of organs or tissues was used to isolate mononuclear immune cells using density gradient centrifugation in Percoll (GE Healthcare, Pittsburgh, PA). The collected immune cells were surface stained with V450 mouse anti-human CD3e and APC mouse anti-human CD56 antibodies (eBioscience, San Diego, CA) to determine the presence of NK-92- EGFR-CAR cells in a specific organ or tissue.

### Statistical analysis

Two-sample *t* test was utilized to compare two independent groups for continuous endpoints if normally distributed. One-way ANOVA was used when three or more independent groups were compared. For non-normally distributed endpoints, such as *in vivo* bioluminescence intensity, a Kruskal-Wallis test was utilized to compare the median of NK-92-EGFR-CAR group to that of NK-92-EV and HBSS-treated groups. For survival data, Kaplan-Meier curves were plotted and compared using a log-rank test. All tests are two-sided. *P* values were adjusted for multiple comparisons using Bonferroni method. *P* values less than 0.05 were considered statistically significant.

## Additional Information

**How to cite this article**: Han, J. *et al.* CAR-Engineered NK Cells Targeting Wild-Type EGFR and EGFRvIII Enhance Killing of Glioblastoma and Patient-Derived Glioblastoma Stem Cells. *Sci. Rep.* **5**, 11483; doi: 10.1038/srep11483 (2015).

## Supplementary Material

Supplementary Information
