# Supplementary material for: CAR-Engineered NK Cells Targeting Wild-Type EGFR and EGFRvIII Enhance Killing of Glioblastoma and Patient-Derived Glioblastoma Stem Cells
Source: Sci Rep. 2015 Jul 9;5:11483. doi: 10.1038/srep11483 (PMC4496728; doi:10.1038/srep11483)
Supplement: Supplementary Information [file srep11483-s1.doc]

**CAR-Engineered NK Cells Targeting Wild-Type EGFR and**

**EGFRvIII Enhance Killing of Glioblastoma and Patient-Derived**

**Glioblastoma Stem Cells**

Jianfeng Han^1,2*^, Jianhong Chu^2*^, Wing Keung Chan^2^, Jianying Zhang^3^, Youwei Wang^2^, Justus B. Cohen^4^, Aaron Victor^2^, Walter H. Meisen^6^, Sung-hak Kim^6^, Paola Grandi^4^, Qi-En Wang^2^, Xiaoming He^5^, Ichiro Nakano^6^, E. Antonio Chiocca^7^, Joseph C. Glorioso III^4^, Balveen Kaur^6^, Michael A. Caligiuri^1,2^, and Jianhua Yu^1,2^

^1^Division of Hematology, Department of Internal Medicine, College of Medicine, The Ohio State University, Columbus, Ohio 43210, USA; ^2^The Ohio State University Comprehensive Cancer Center, Columbus, Ohio 43210, USA; ^3^Center for Biostatistics, The Ohio State University, Columbus, Ohio 43210, USA; ^4^Department of Microbiology and Molecular Genetics, University of Pittsburgh School of Medicine, Pittsburgh, PA, USA. ^5^Department of Biomedical Engineering, The Ohio State University, Columbus, Ohio 43210, USA; ^6^Department of Neurological Surgery, The Ohio State University, Comprehensive Cancer Center, Columbus, Ohio 43210, USA; ^7^Department of Neurosurgery, Brigham and Women’s Hospital, Harvard Medical School, Boston, Massachusetts 02115, USA

**Correspondence and requests for materials should be addressed to Dr. Jianhua Yu,** Division of Hematology, Department of Internal Medicine, The Ohio State University, Biomedical Research Tower 816, 460 West 12^th^ Avenue, Columbus, OH 43210, USA; Phone: (614)-293-4157; Fax: (614)-688-4028; Email address: [jianhua.yu@osumc.edu](mailto:jianhua.yu@osumc.edu)

***These authors contributed equally to this work.**

**Supplemental Figure 1.** **EGFR-CAR primary NK cells display enhanced killing of EGFR^+^ GB cells and patient-derived GB stem cells.** (**A**) EGFR-CAR-modified primary NK cells (primary NK-CAR) displayed augmented cytolytic activity towards EGFR^+^ Gli36dEGFR and U251 GB cell lines in comparison with mock-transduced primary NK cells (primary NK-EV). (**B**) EGFR-CAR-modified NK cell (primary NK-CAR) showed enhanced cytotoxicity towards EGFR^+^-patient-derived GB30 and GB157V3SL stem cells in comparison with mock-transduced primary NK cells (primary NK-EV). Data presented are representative of three experiments with similar results, examining NK cells isolated from different healthy donors. **p* < 0.05; ***p* < 0.01.

**Supplemental Figure 2. Enhanced target recognition of NK-92-EGFR-CAR cells depends on expression of EGFR on cell surface.** (**A**) Flow cytometric analysis using anti-EGFR antibody (solid line) or IgG isotype control (dotted line) of 293T cells transduced with empty vector (EV; left), wtEGFR (center) or EGFRvIII (right). (**B**) Cytotoxicity of NK-92-EV or NK-92-EGFR-CAR (top panel) and NKL-EV or NKL-EGFR-CAR (lower panel) against 293T-EV (left), 293T-EGFR (center), and 293T-EGFRvIII (right) cells. 293T cells were incubated with NK cells at various Effector/Target (E/T) ratios for 4 h. Tumor lysis was determined using chromium-51 release assay. (**C**) After coincubation of target cells and effector cells for 24 h, supernatants from the co-cultures were measured for IFN-γ secretion using ELISA. Data presented are representative of three experiments with similar results. **p* < 0.05; ***p* < 0.01.

**Supplemental Figure 3. The effects of NK-92-EGFR-CAR cells are blunted by an EGFR blocking antibody (Ab).** (**A**) Cytotoxicity of NK-92-EV or NK-92-EGFR-CAR against GB30 cells (Left) or U251 cells (Right) pretreated with an EGFR-specific monoclonal antibody 528 or an IgG-matched isotype control antibody. Target cells were incubated with pre-treated NK-92-EV or NK-92-EGFR-CAR cells at various Effector/Target (E/T) ratios for 4 h. Tumor lysis was determined using a chromium-51 release assay. (**B**) After co-incubation of target cells and effector cells for 24 h, supernatants from the co-cultures were measured for IFN-γ secretion using ELISA. Data presented are representative of three experiments with similar results. **p* < 0.05; ***p* < 0.01. Ab = EGFR-specific monoclonal antibody 528; iso= IgG-matched isotype control antibody.

**Supplemental Figure 4. NK-92-EGFR-CAR cells are located in tumor area after intratumoral injection.**  NK-92-EGFR-CAR cells were intratumorally injected into mouse brains seven days after GB30 implantation. The mouse brains were harvested 3 days later, fixed by formalin, embedded by paraffin, and processed for Hematoxylin and Eosin (H&E) staining. H&E staining showed that NK-92-EGFR-CAR cells only existed inside tumor area. Magnifications of 20x, 40x, 100x, and 200x were shown (Objective: 2×, 4×, 10× or 20×; Eyepiece: 10×). Dashed arrows indicate the GB30 tumor area. Solid arrows indicate the NK-92-EGFR-CAR cell area.

**Supplemental Figure 5. NK-92-EGFR-CAR cells cause negligible damage to the brain cells.**  NK-92-EGFR-CAR cells were intracranially injected into mouse brains. The injected and control mouse brains were harvested 3 days later, fixed by formalin, embedded by paraffin, and processed for cleaved caspase-3 immunohistochemistry (IHC) staining. IHC staining showed that majority of apoptotic cells seem to be NK-92-EGFR-CAR cells (A) and some host cells in the tissue damaged by a needle injection (B). Nearly no damages by NK-92-EGFR-CAR cells were observed in other areas (C, D) when compared with brain tissues harvested from un-treated control mice (E). Magnifications of 100×, and 200× were shown (Objective: 10× or 20×; Eyepiece: 10×). The solid arrow indicates the needle track area. The dashed arrow indicates the area where NK-92-EGFR-CAR cells reside.

**Supplementary Material and Methods**

**Generation of 293T cell lines stably expressing wtEGFR or EGFRvIII.** Phoenix cells were co-transfected with the pBABE-wtEGFR (wtEGFR expression construct), pBABE-EGFRvIII (EGFRvIII expression construct), or pBABE empty vector together with Sara3 packaging plasmid using calcium phosphate transfection reagent (Promega, Madison, WI, USA). Two days after transfection, supernatants were harvested to infect 293T cells in the presence of polybrene (8 μg/mL). GFP positive cells were sorted using a FACS Aria II cell sorter (BD Biosciences, San Jose, CA, USA).

**Lentivirus production and transduction of primary NK cells.** Lentiviruses were produced as previously described[^1^](#_ENREF_1) and were concentrated by ultracentrifuge at 20,000g for 90 minutes at 4°C and resuspended with 1× PBS. Human primary NK cells were isolated from peripheral blood leukopacks of healthy donors (American Red Cross, Columbus, Ohio) as described previously [^2^](#_ENREF_2) and infected with lentiviruses (MOI = 2.5) by three consecutive rounds of centrifugation at 2000 rpm and 32 °C for 2 h (gentle resuspension between rounds), and GFP positive cells were sorted using a FACS Aria II cell sorter (BD Biosciences, San Jose, CA, USA). Standard 4-h ^51^Cr release assays were performed to evaluate the cytotoxicity of primary NK cells transduced with EGFR-CAR plasmid or control vector against U251 and Gli36dEGFR GB cell lines and GB30 and GB157V3SL patient-derived GB stem cells.

**Antibody blocking assay.** GB30 stem cells and U251 cell line were pretreated with 10 μg/ml EGFR neutralizing antibody (clone 528, EMD Millipore, Billerica, MA) or an isotype-matched antibody at 37°C for 30 minutes. Standard 4-h ^51^Cr release assays were then performed to evaluate cytotoxicity of NK-92-EGFR-CAR cells and control cells against the 528 antibody- or IgG- pretreated target cells. IFN-γ secretion was also quantified by ELISA on cell-free supernatants after 1 × 10^6^ target cells pretreated with 528 antibody or IgG were incubated with an equal number of NK-92-EGFR-CAR cells or control cells in 96-well V bottom plates for 24 h.

**Hematoxylin and Eosin (H&E) staining of brain sections of GB30-bearing mice.** NSG mice were intracranially injected with 5 × 10^4^ GB30 cells on day 0. On day 7, the mice were intracranially injected with 2 × 10^6^ NK-92-EGFR-CAR cells in 5 μl HBSS. On day 10, the mice were sacrificed and brain tissues were harvested and immersed in 10% formalin for 24 h, and then brains were embedded in paraffin and processed for H&E staining.

**Cleaved Caspase3 immunohistochemistry (IHC) staining.** NSG mice were intracranially injected with 2 × 10^6^ NK-92-EGFR-CAR cells in 5 μl HBSS. On day 3, the injected mice and the control mice were sacrificed and brain tissues were harvested, immersed in 10% formalin for 24 h, and then were embedded and processed for cleaved Caspase-3 IHC staining using a cleaved Caspase-3 (5A1) rabbit mAb (Cell Signaling Technology, Danvers, MA).

**References:**

1 Chu, J. *et al.* CS1-specific chimeric antigen receptor (CAR)-engineered natural killer cells enhance in vitro and in vivo antitumor activity against human multiple myeloma. *Leukemia* **28**, 917-927, doi:10.1038/leu.2013.279 (2014).

2 He, S. *et al.* MicroRNAs activate natural killer cells through Toll-like receptor signaling. *Blood* **121**, 4663-4671, doi:10.1182/blood-2012-07-441360 (2013).
